# Supplementary material for: Foxa2 attenuates steatosis and inhibits the NF-κB/IKK signaling pathway in nonalcoholic fatty liver disease
Source: PeerJ. 2023 Dec 7;11:e16466. doi: 10.7717/peerj.16466 (PMC10710773; doi:10.7717/peerj.16466)

Figure 1

C

Foxa2

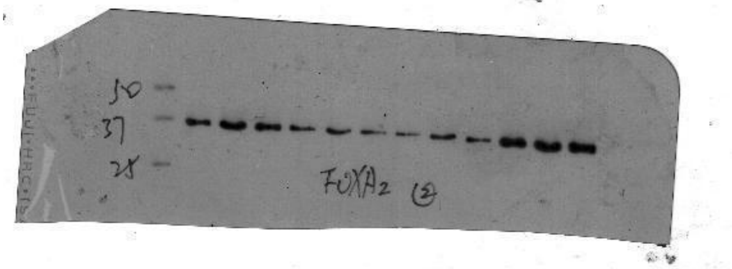

GAPDH

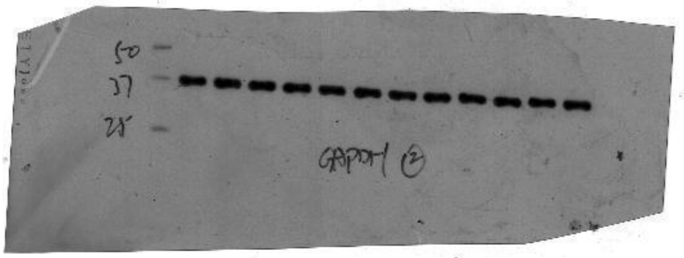

Figure 3

FAS

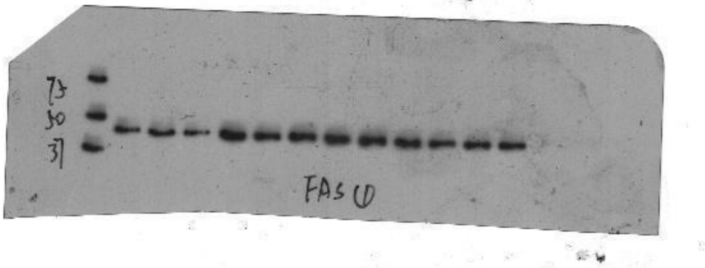

ACC

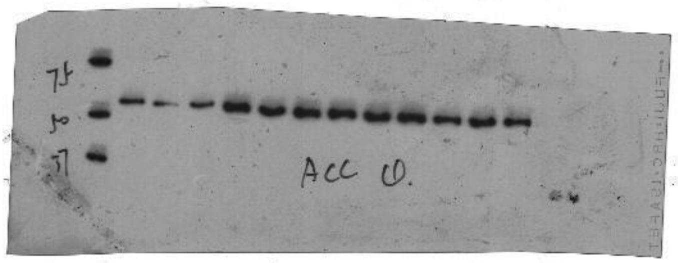

CPT1α

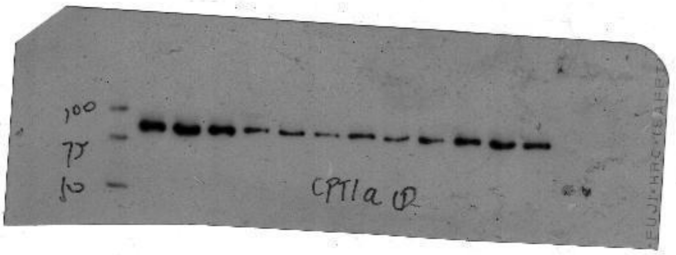

GAPDH

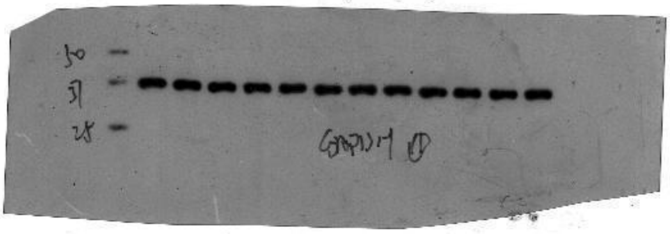

Figure 4

A

Foxa2

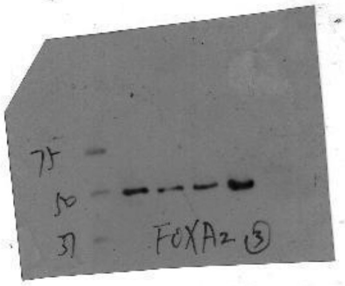

GAPDH

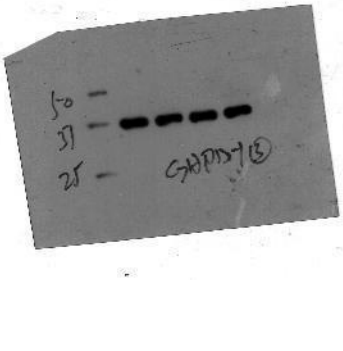

E

FAS

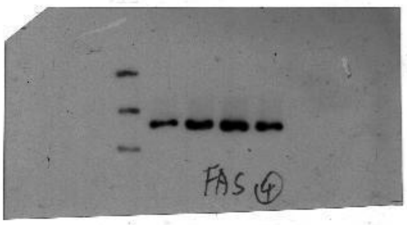

ACC

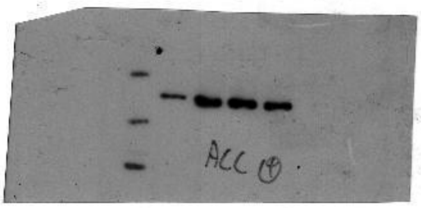

CPT1α

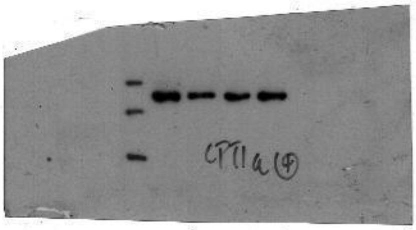

GAPDH

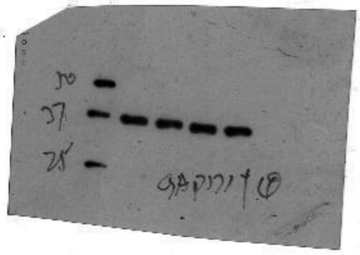

Figure 5

A

Foxa2

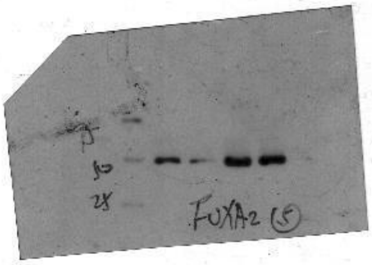

GAPDH

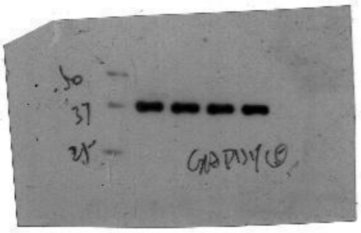

B

p-NF-κB

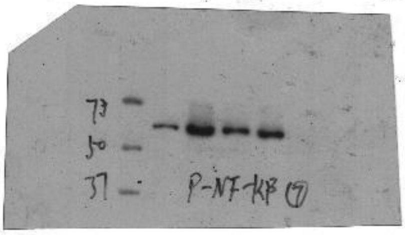

NF-κB

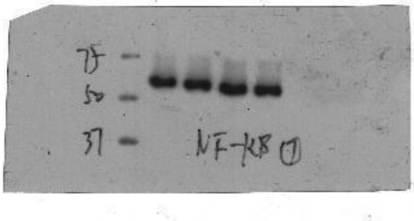

p-IKK

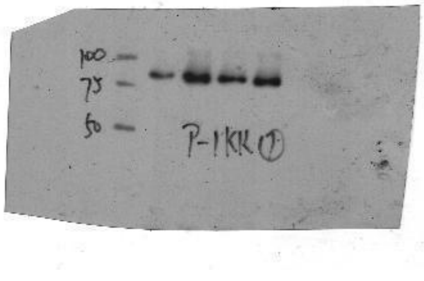

IKK

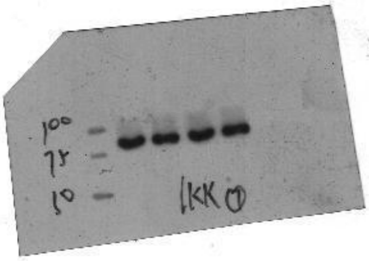

GAPDH

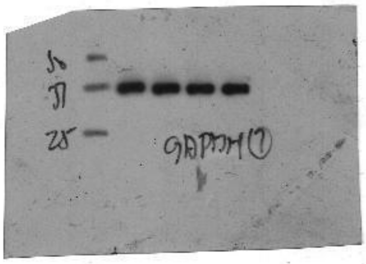

C

FAS

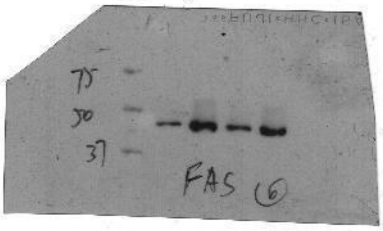

ACC

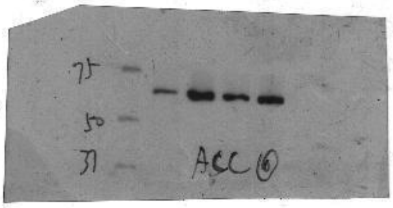

CPT1α

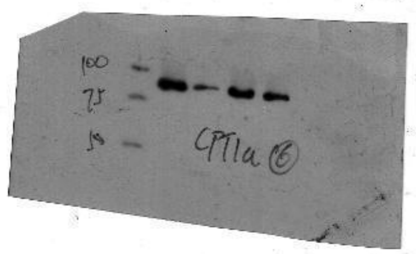

GAPDH

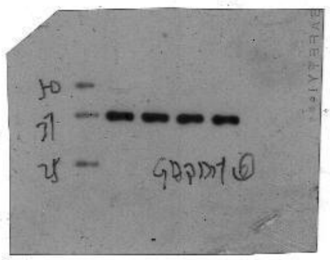

Supplement: Supplemental Information 3 [file peerj-11-16466-s003.pdf]
