# Supplementary figures and images for: Foxa2 attenuates steatosis and inhibits the NF-κB/IKK signaling pathway in nonalcoholic fatty liver disease
Source: PeerJ. 2023 Dec 7;11:e16466. doi: 10.7717/peerj.16466 (PMC10710773; doi:10.7717/peerj.16466)

C

Foxa2

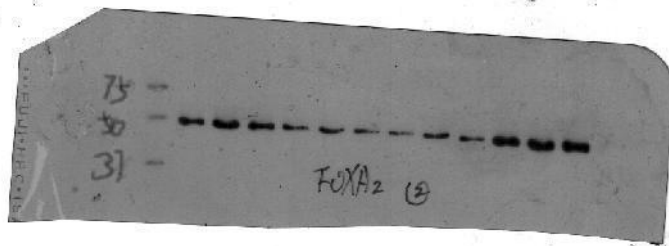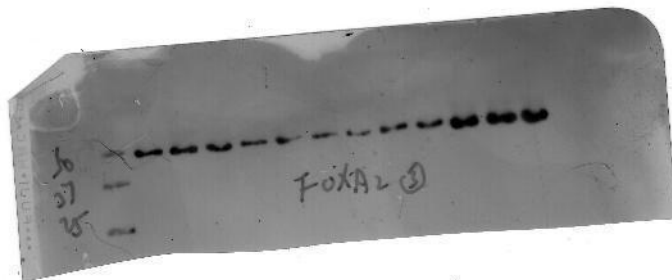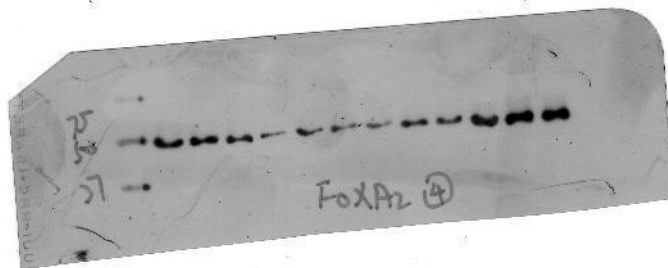

GAPDH

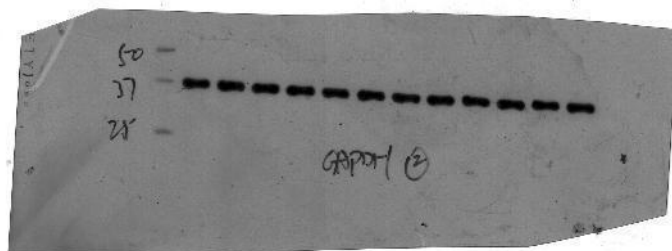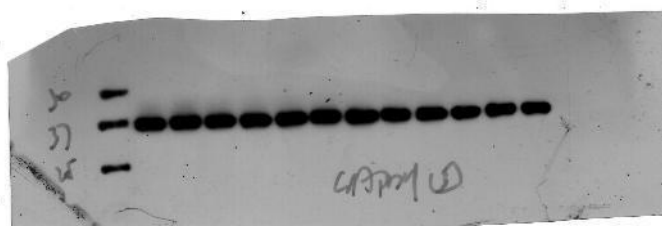

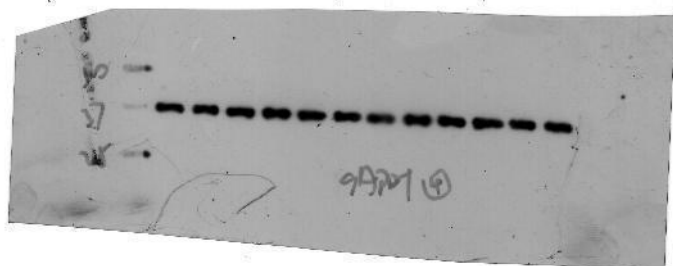

Supplement: Supplemental Information 4 [file peerj-11-16466-s004.zip › Raw data/Figure 1.pdf]

**C**

**HE**

Control

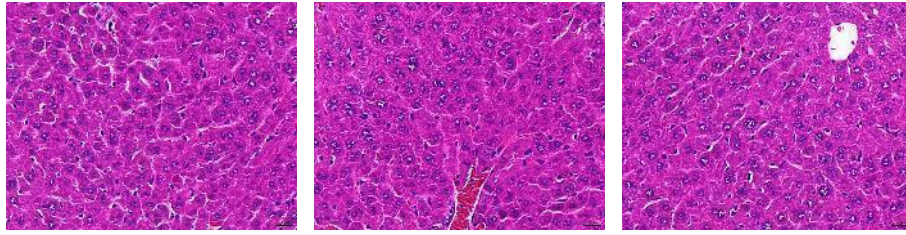

HFD

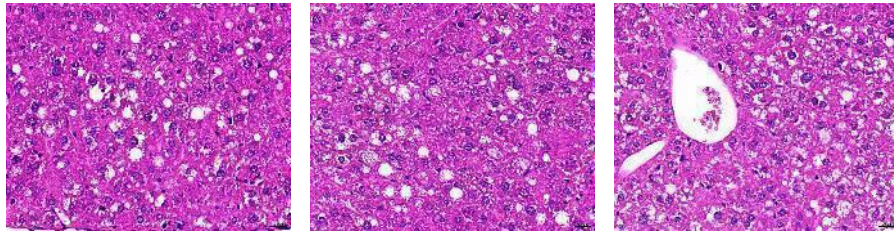

HFD + oe-NC

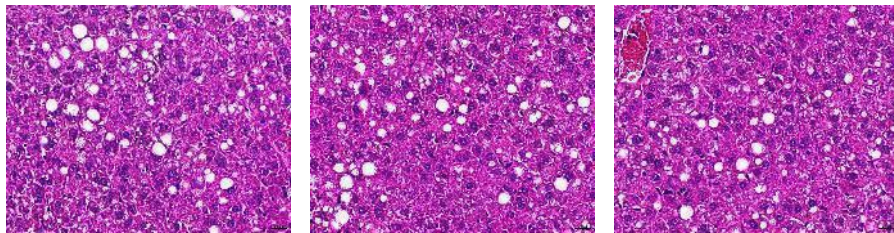

HFD + oe-Foxa2

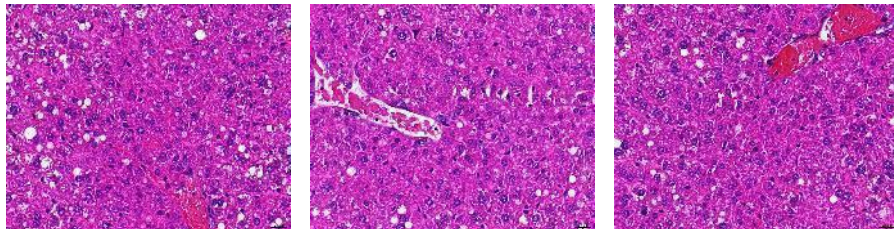

## Oil Red O

Control

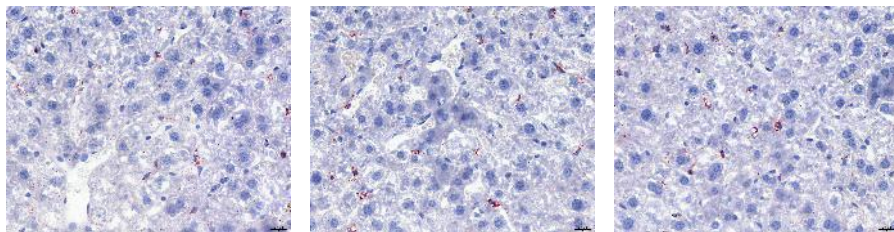

HFD

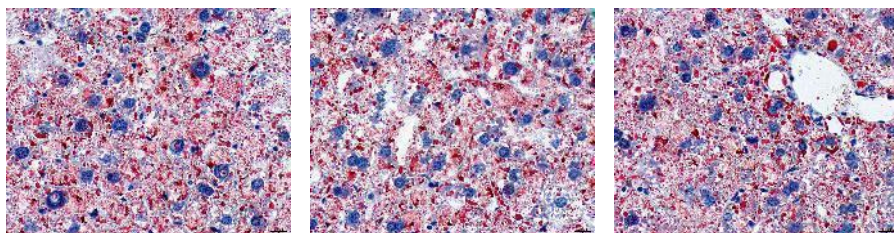

HFD + oe-NC

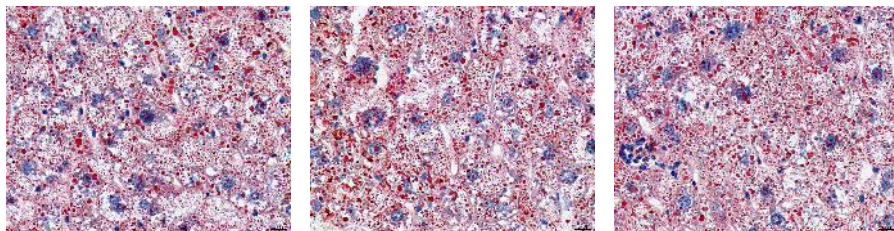

HFD + oe-Foxa2

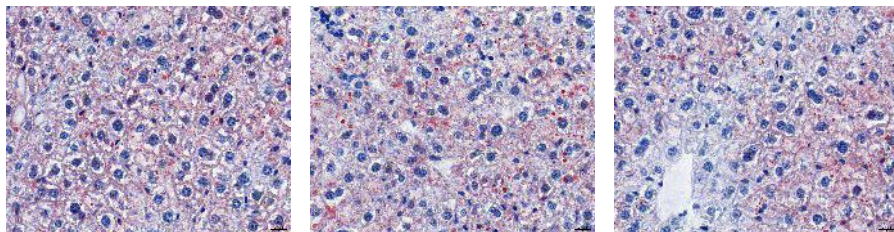

Supplement: Supplemental Information 4 [file peerj-11-16466-s004.zip › Raw data/Figure 2.pdf]

FAS

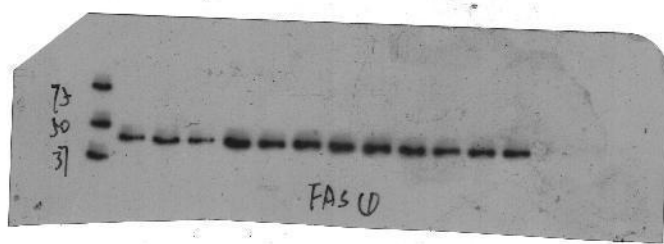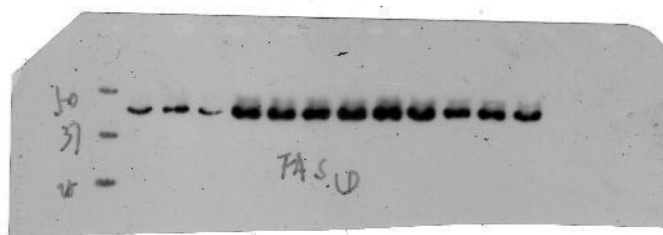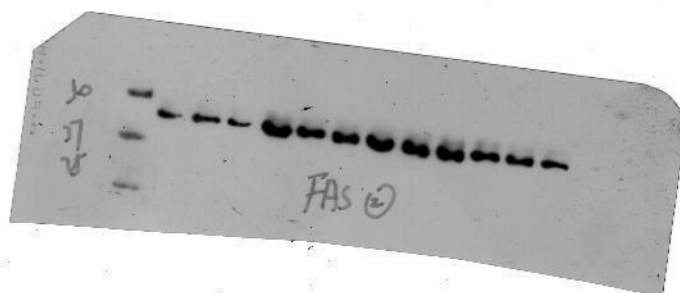

ACC

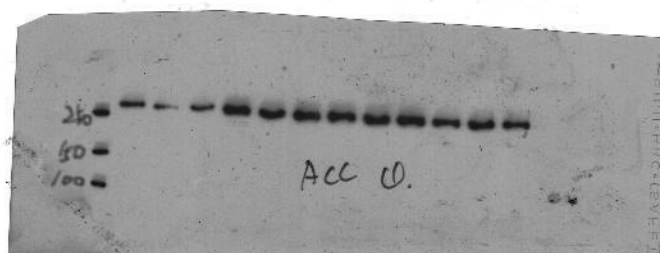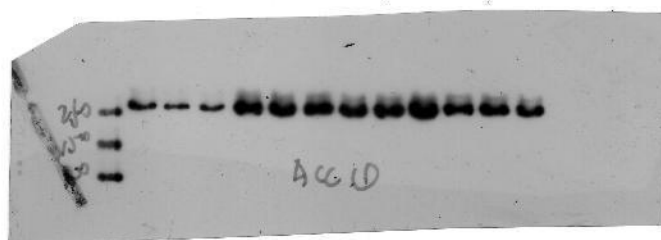

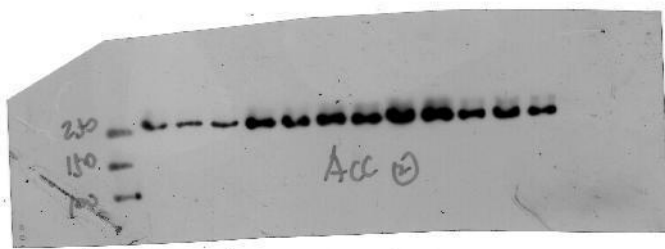

CPT1a

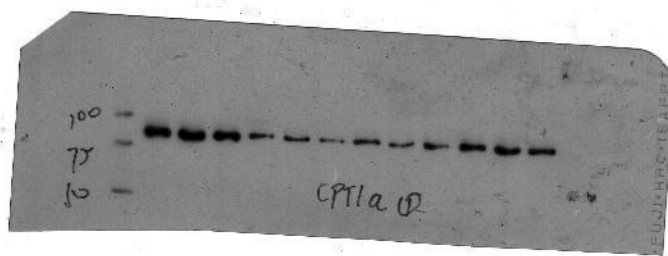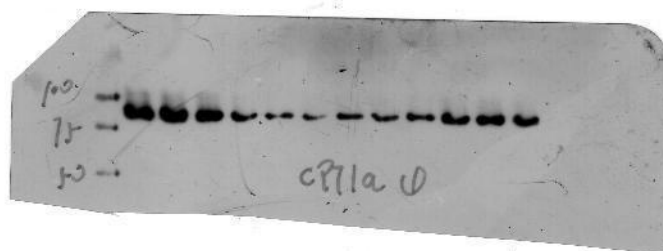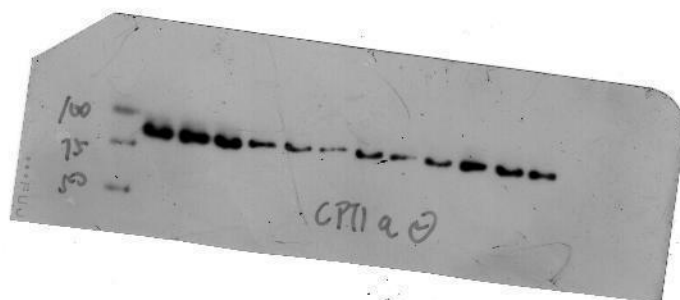

GAPDH

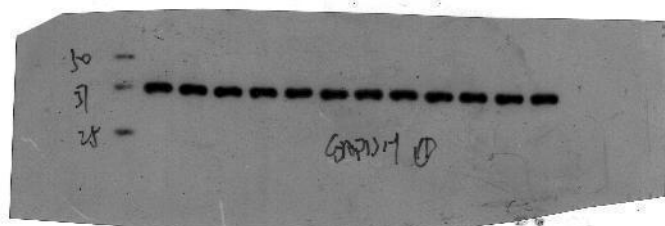

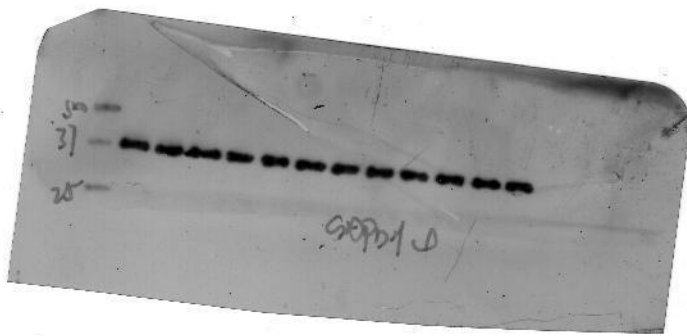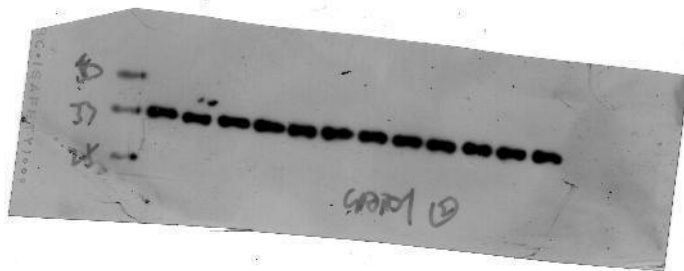

Supplement: Supplemental Information 4 [file peerj-11-16466-s004.zip › Raw data/Figure 3.pdf]

**A**

Foxa2

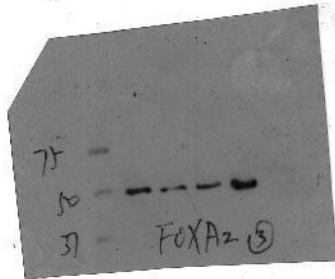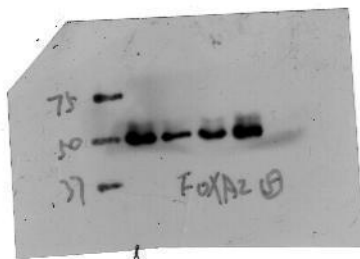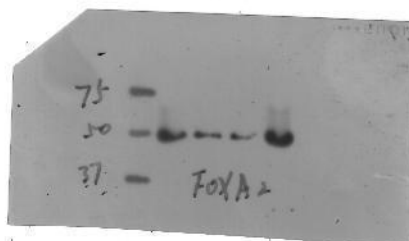

GAPDH

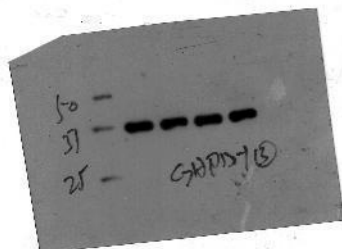

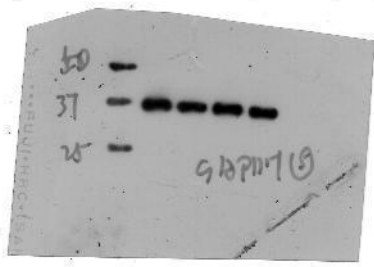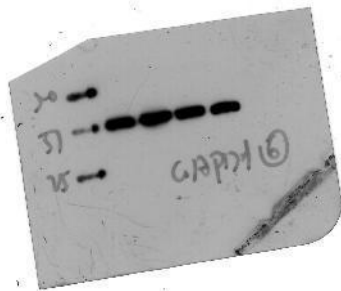

**C**

Control

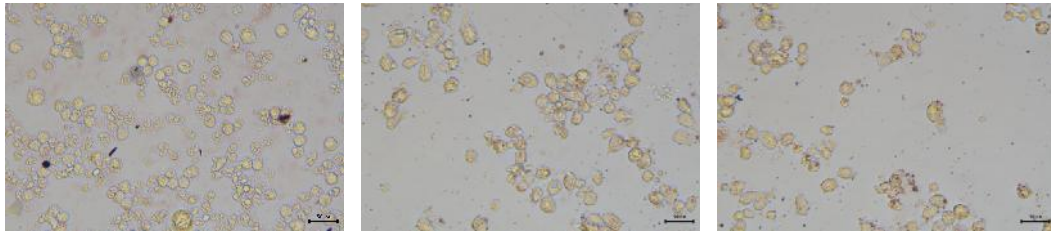

OA

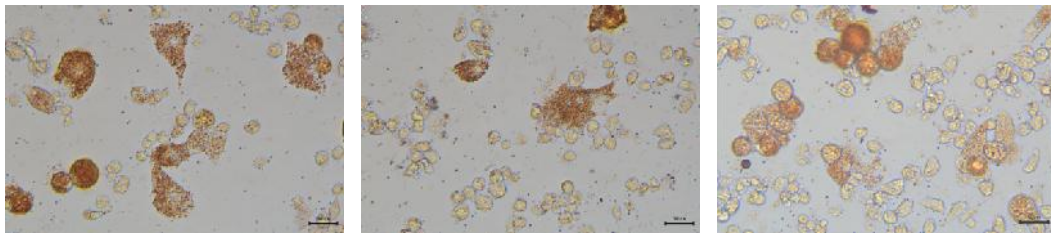

OA + oe-NC

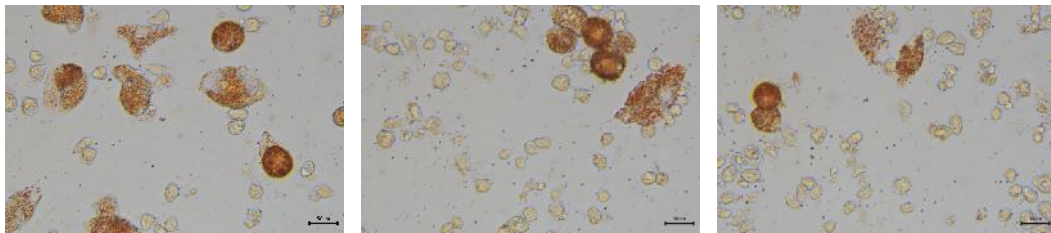

OA + oe-Foxa2

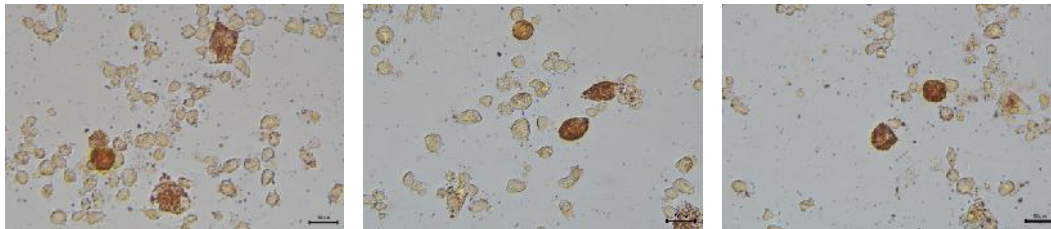

**E**

FAS

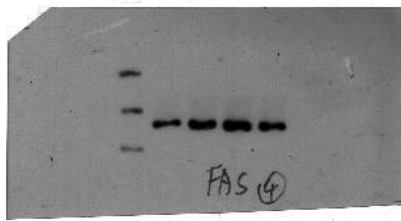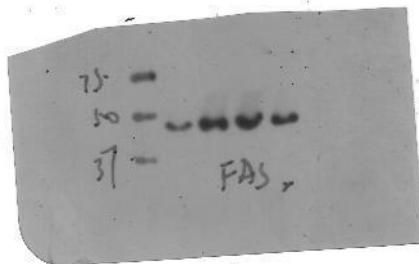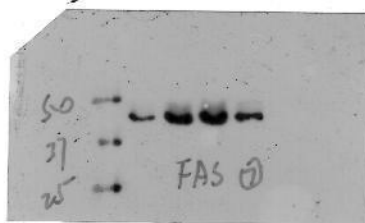

ACC

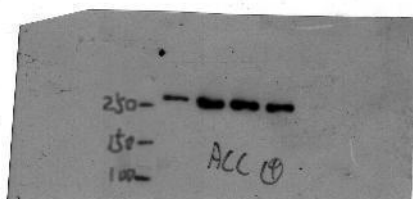

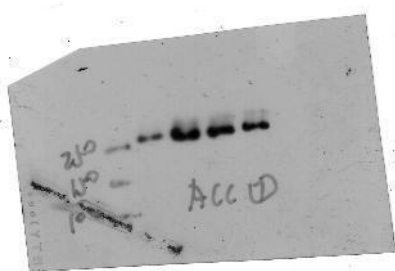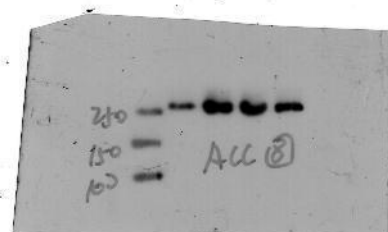

CPT1 $\alpha$

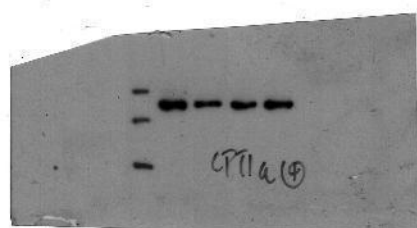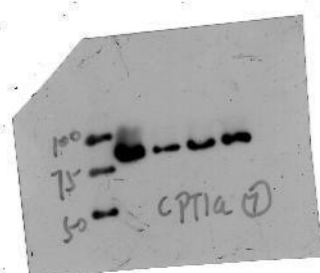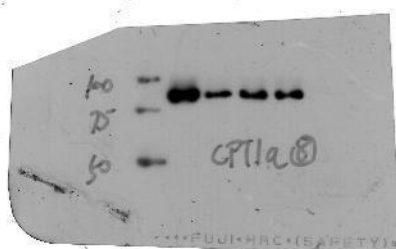

# GAPDH

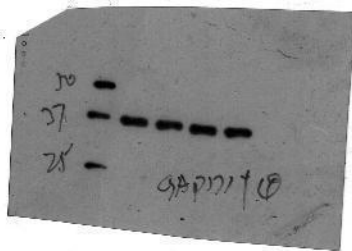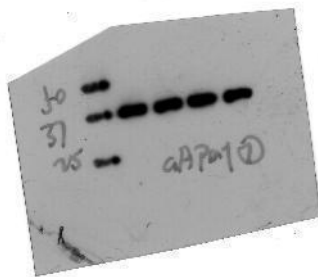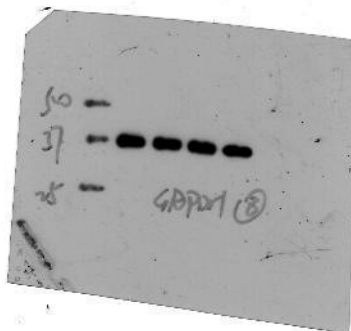

Supplement: Supplemental Information 4 [file peerj-11-16466-s004.zip › Raw data/Figure 4.pdf]

**A**

Foxa2

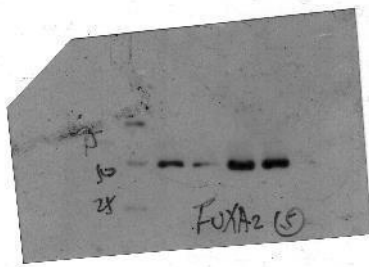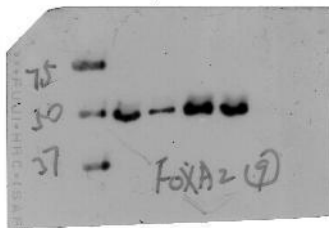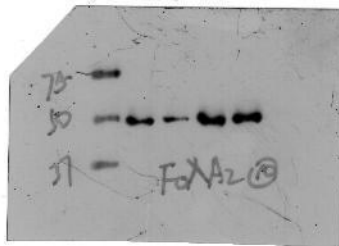

GAPDH

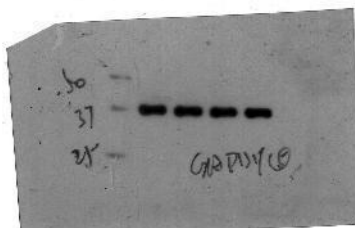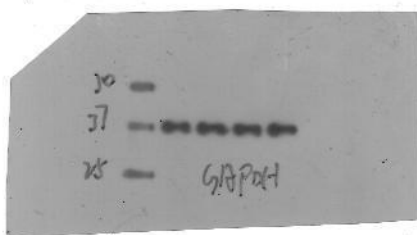

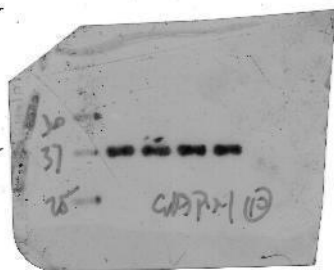

## B

p-NF- $\kappa$ B

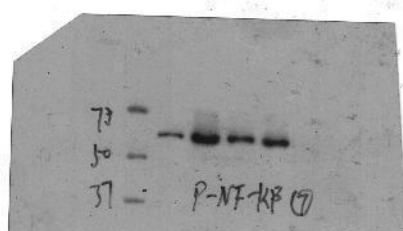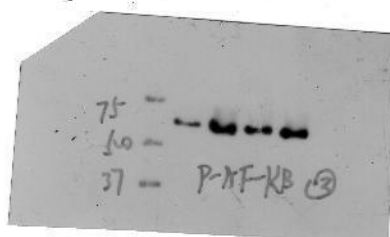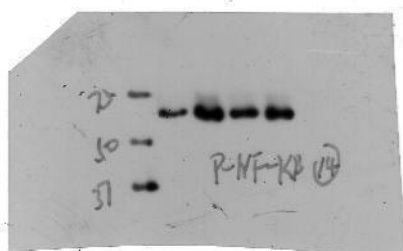

NF- $\kappa$ B

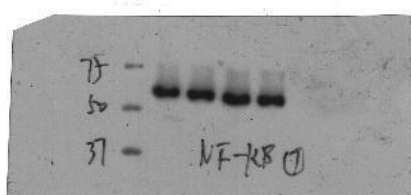

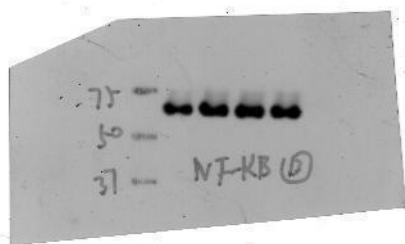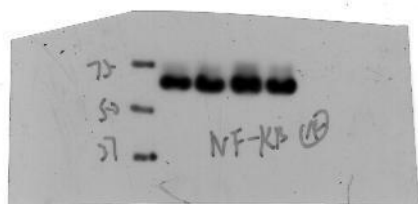

p-IKK

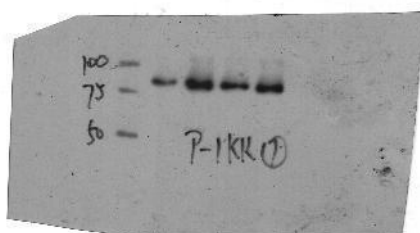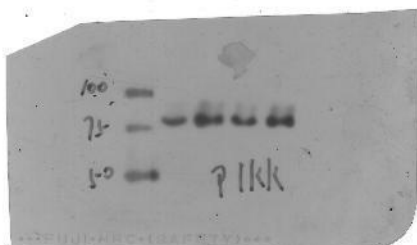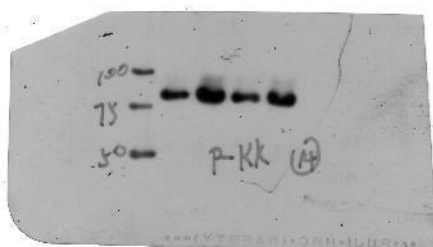

IKK

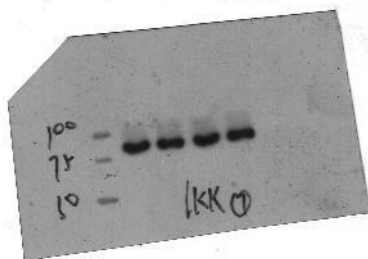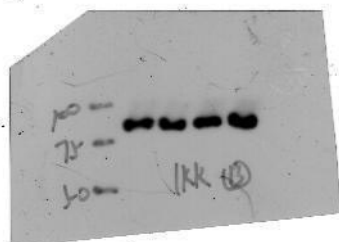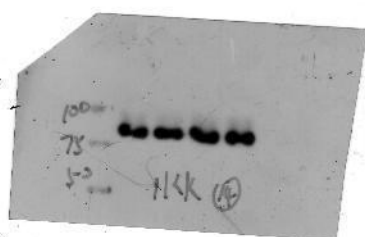

GAPDH

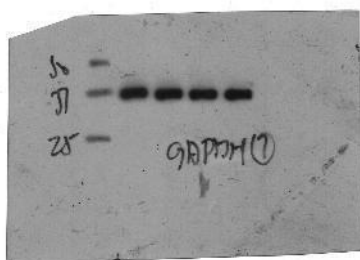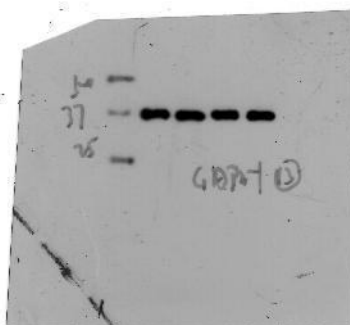

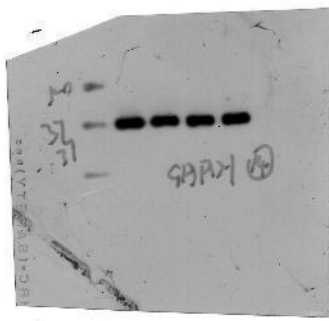

**C**

FAS

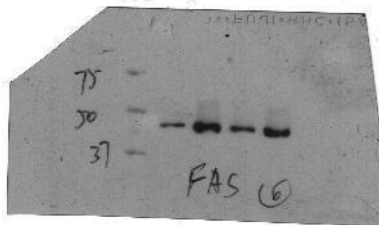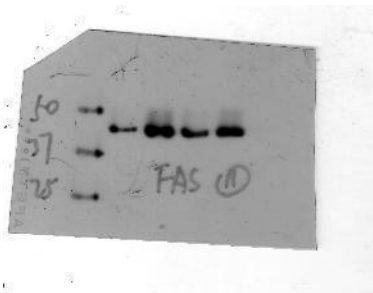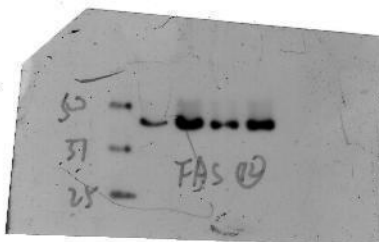

ACC

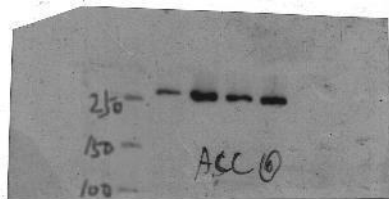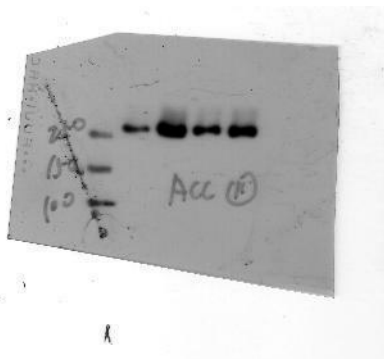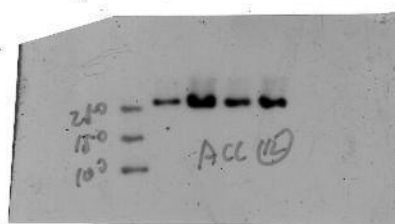

CPT1 $\alpha$

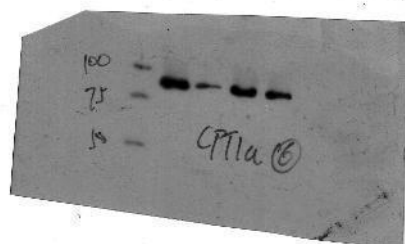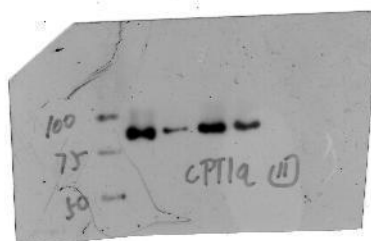

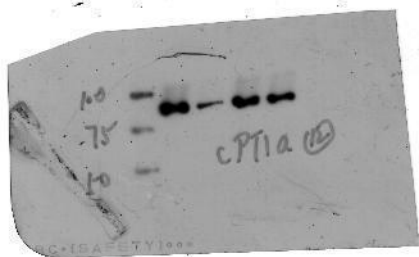

GAPDH

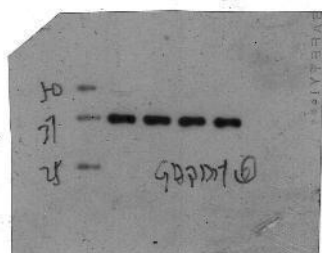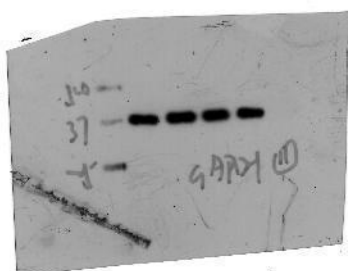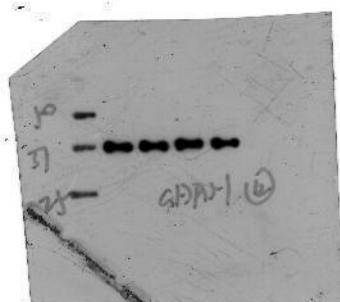

Supplement: Supplemental Information 4 [file peerj-11-16466-s004.zip › Raw data/Figure 5.pdf]
