# Supplementary material for: Foxa2 attenuates steatosis and inhibits the NF-κB/IKK signaling pathway in nonalcoholic fatty liver disease
Source: PeerJ. 2023 Dec 7;11:e16466. doi: 10.7717/peerj.16466 (PMC10710773; doi:10.7717/peerj.16466)
Supplement: Supplemental Information 5 [file peerj-11-16466-s005.pdf]

# Figure 1

# C

Foxa2

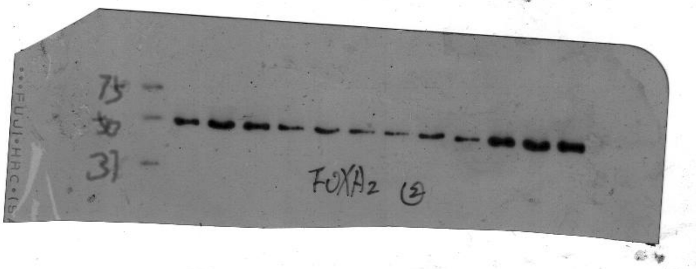


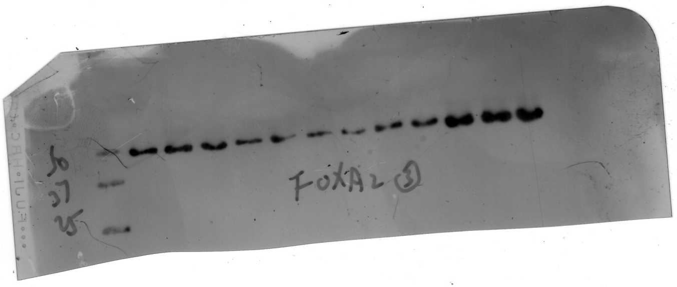


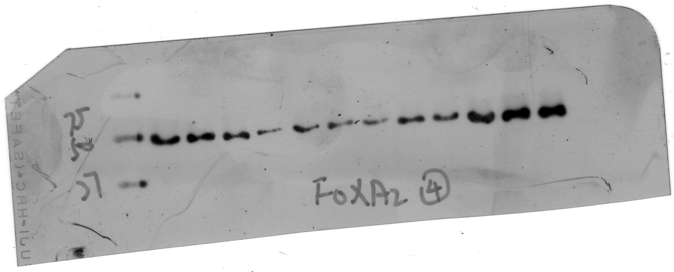


GAPDH

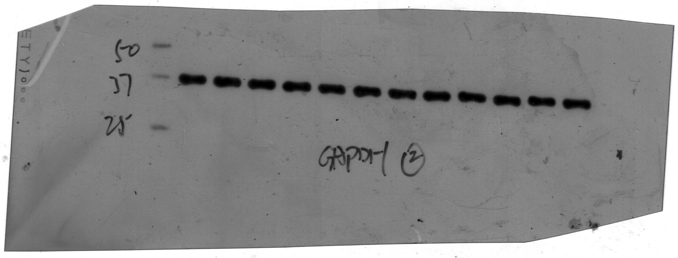

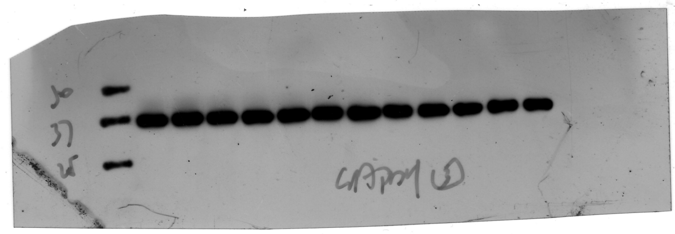

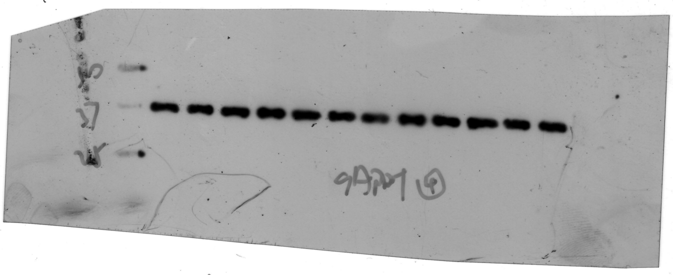

# Figure 3

FAS

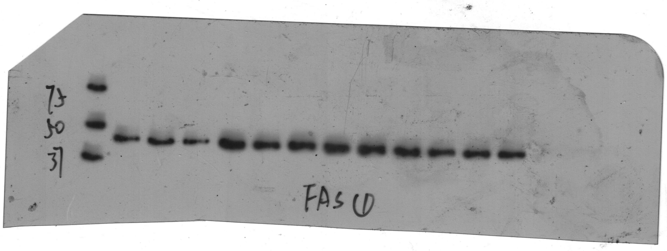

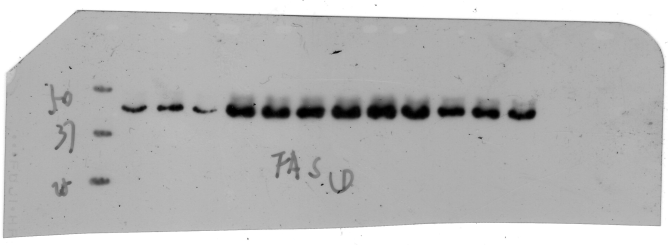

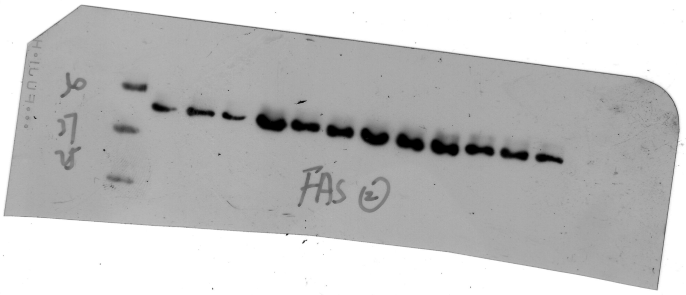


ACC

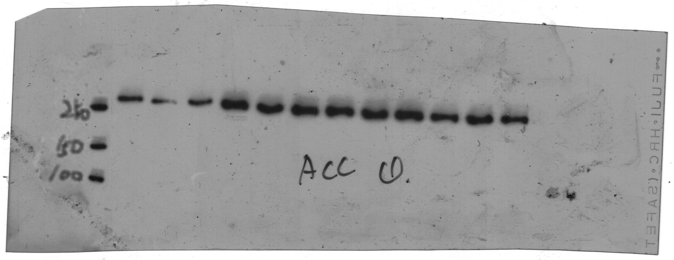


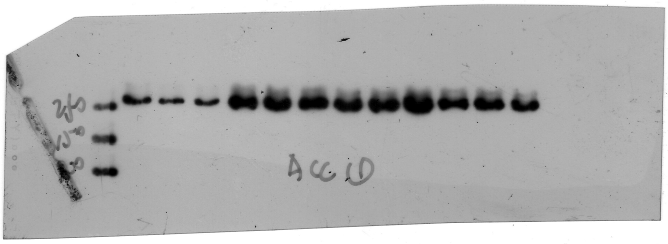


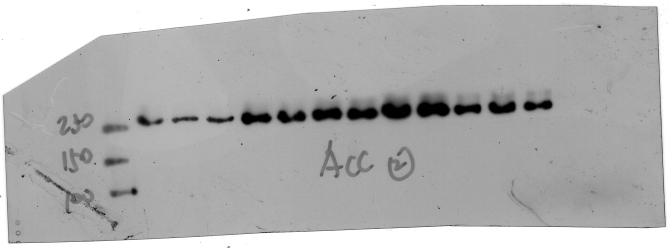


CPT1a

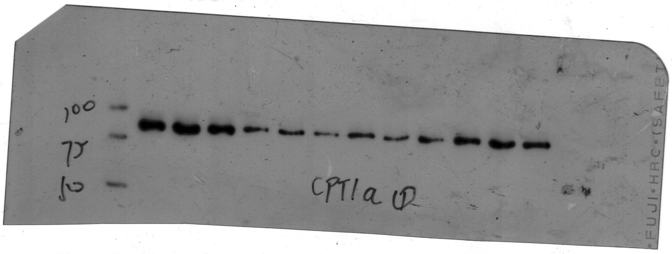

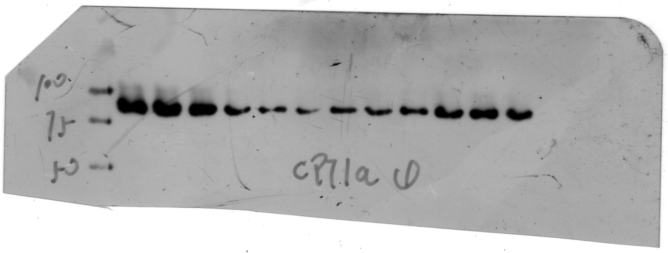


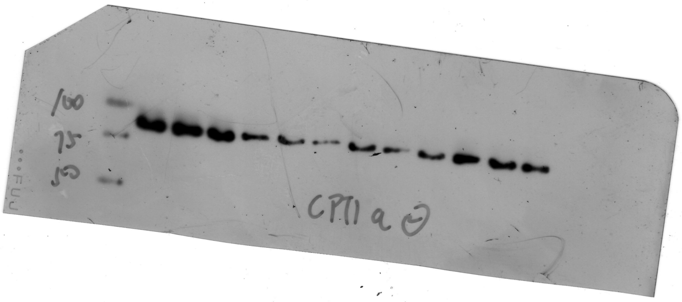


GAPDH

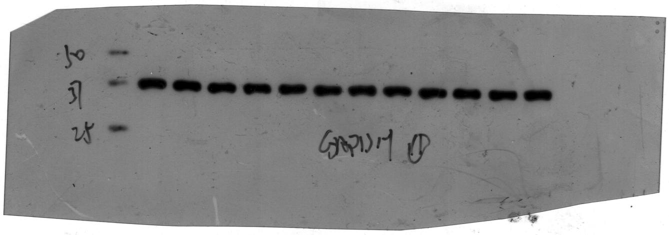


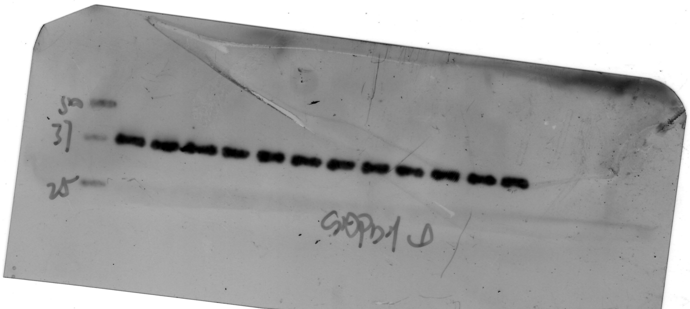


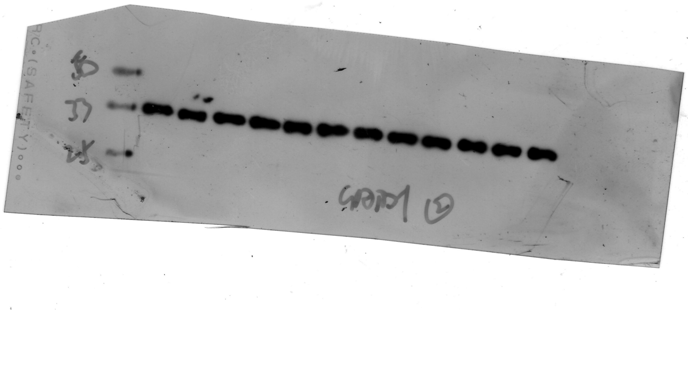

# Figure 4

## A

Foxa2

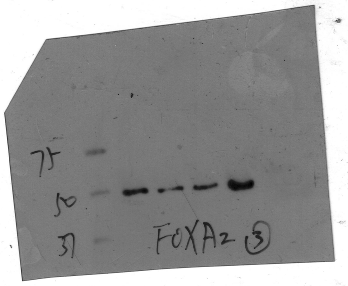


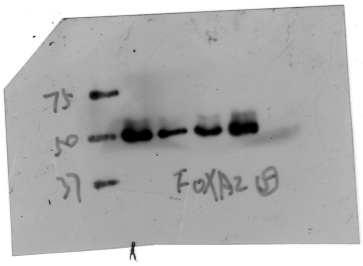


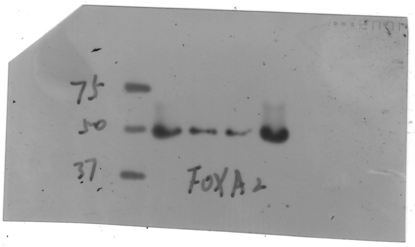


GAPDH

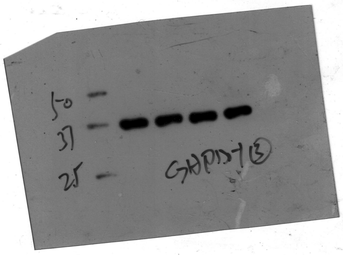

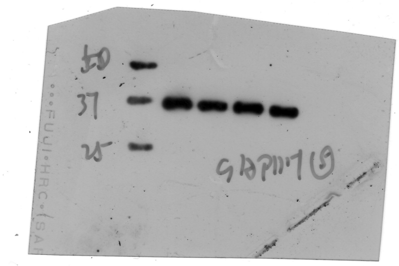


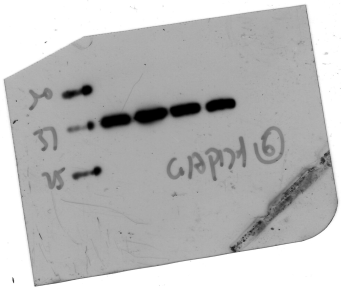


# E

FAS

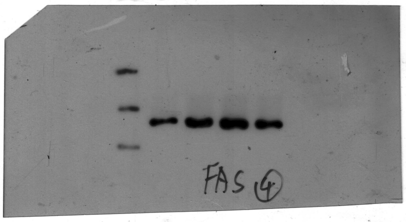


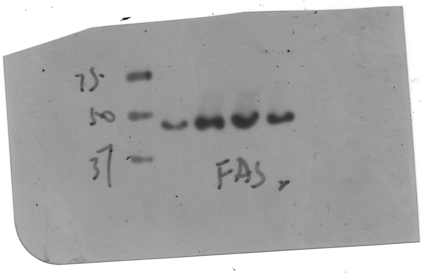

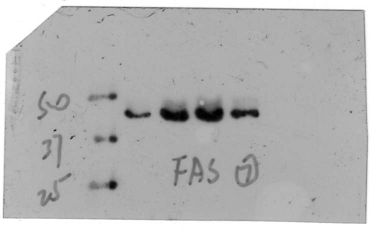


ACC

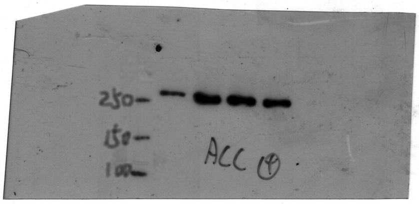


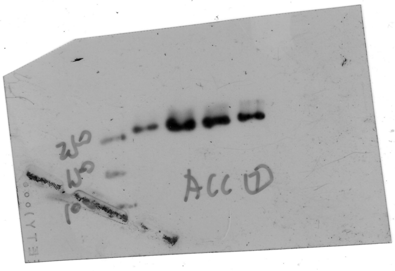


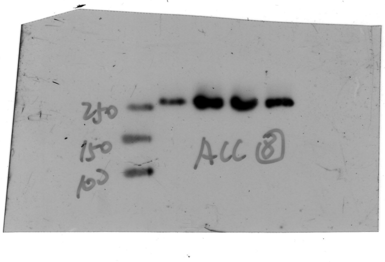


CPT1α

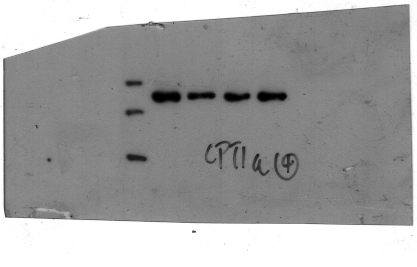

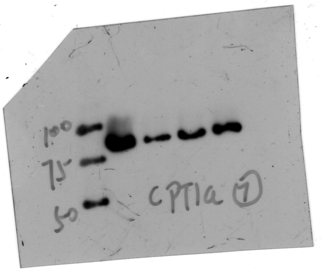
100
75
50
CPT1a ⑦

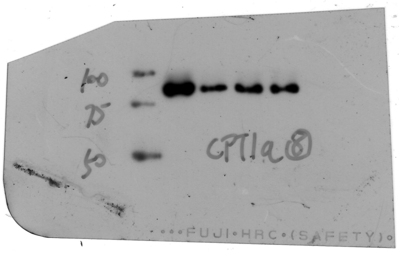
100
75
50
CPT1a ⑧
FUJI • HRC • (SAFETY)

GAPDH

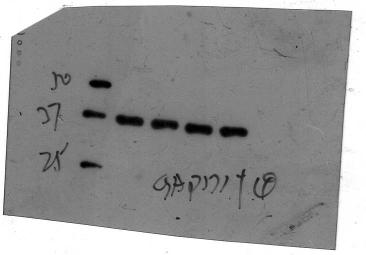
50
37
25

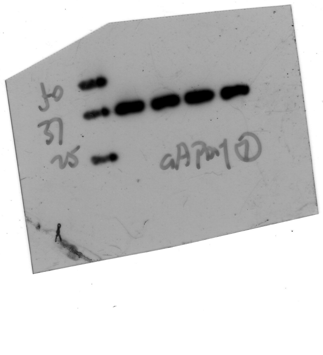
50
37
25

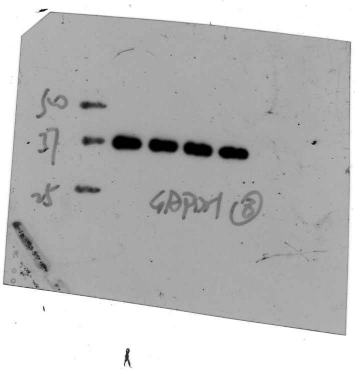


# Figure 5

## A

Foxa2

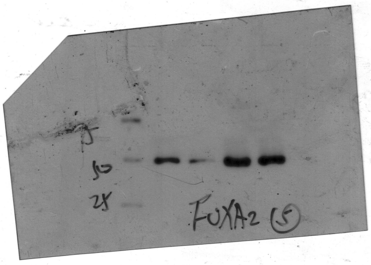


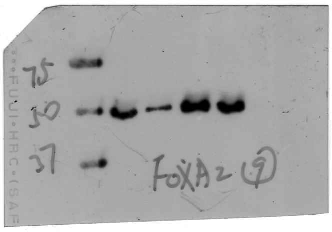


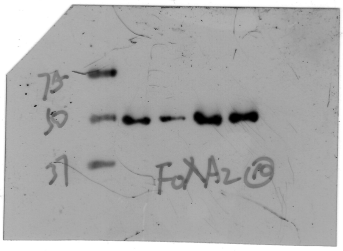


GAPDH

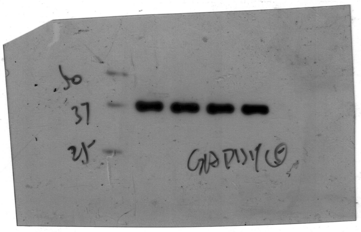
50
37
25
GAPDH

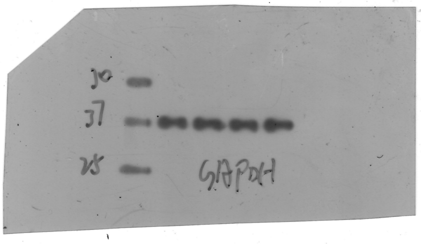
50
37
25
GAPDH

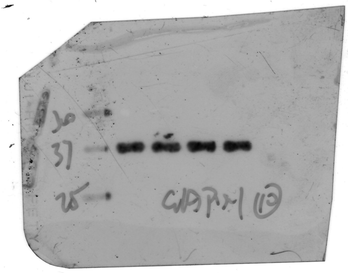
50
37
25
GAPDH

## B

p-NF-κB

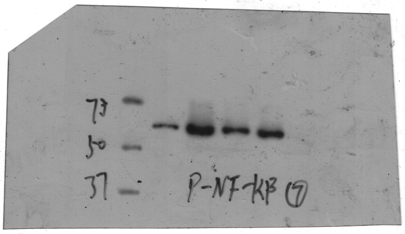
75
50
37
P-NF-KB

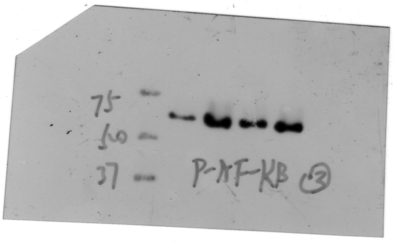
75
50
37
P-NF-KB ③

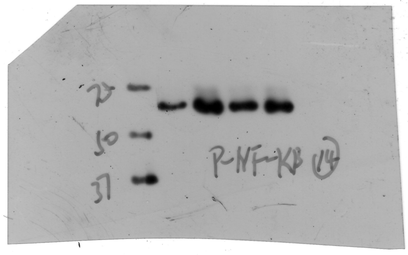
75
50
37
P-NF-KB

NF-κB

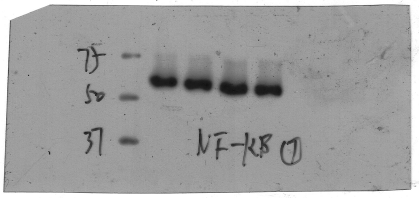
75
50
37
NF-KB

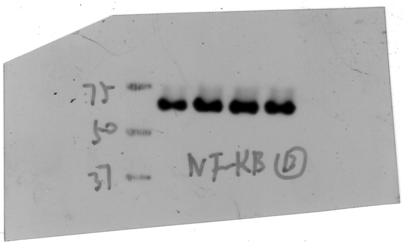
75
50
37
NF-KB

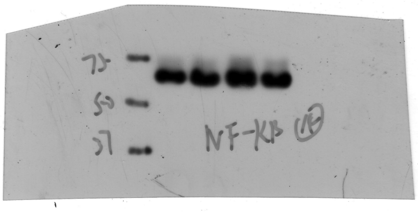
75
50
37
NF-KB

p-IKK

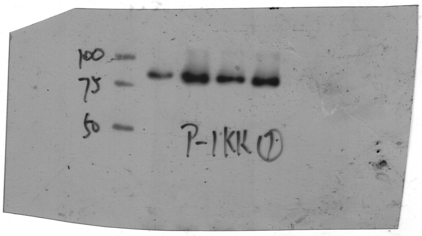
100
75
50
P-IKK

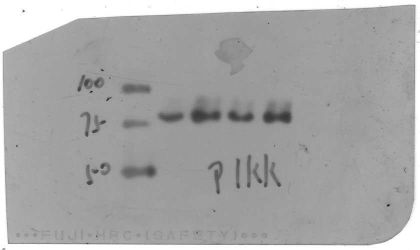
100
75
50
P IKK

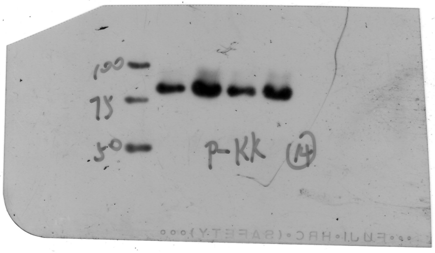
100
75
50
P-IKK

IKK

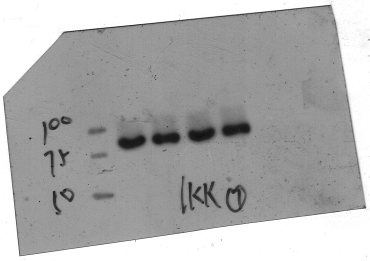
100
75
50
IKK

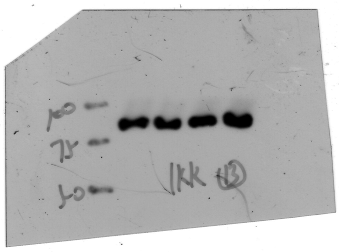
100
75
50
IKK

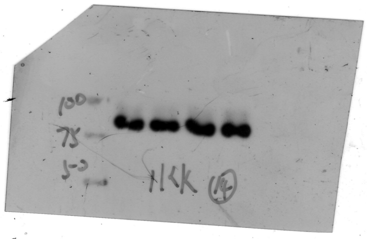
100
75
50
IKK

GAPDH

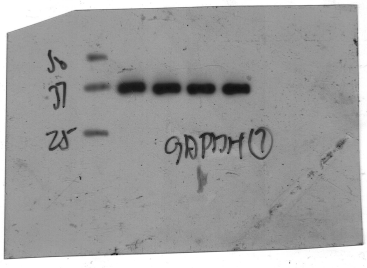


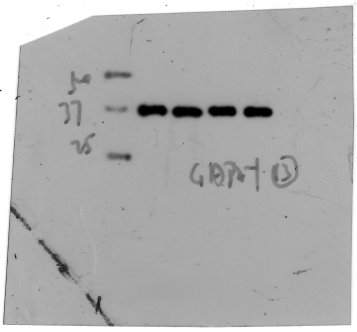


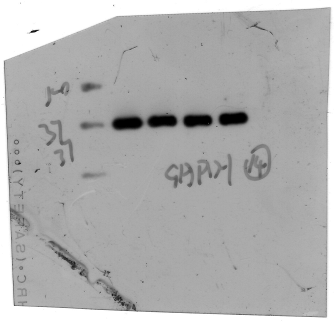

# C

FAS

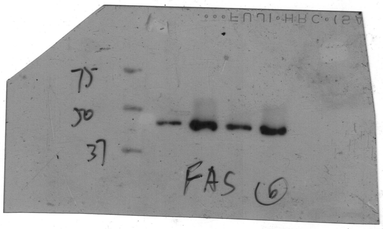

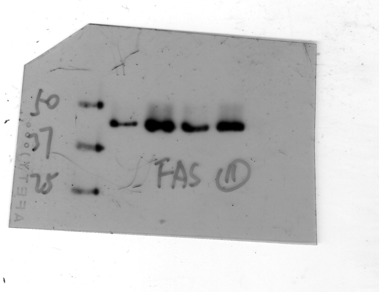


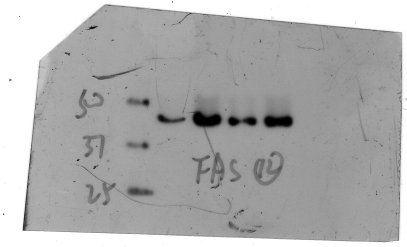


ACC

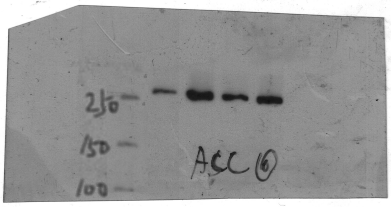


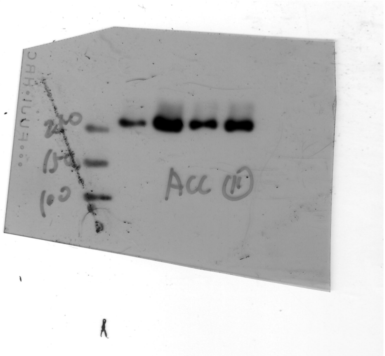


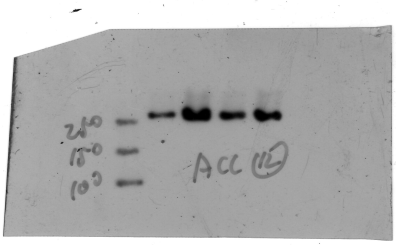


CPT1α

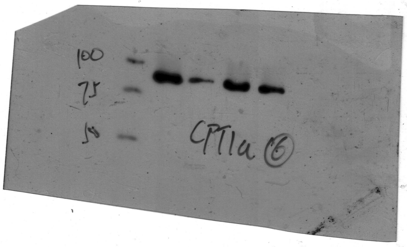


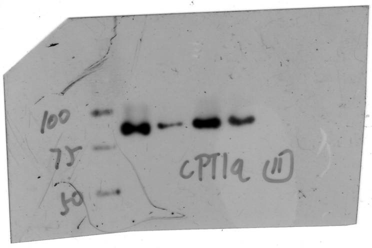


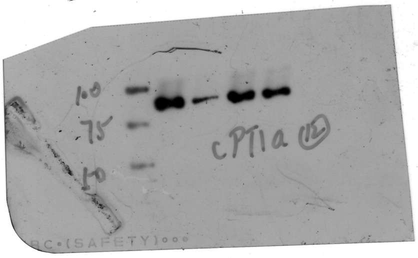


GAPDH

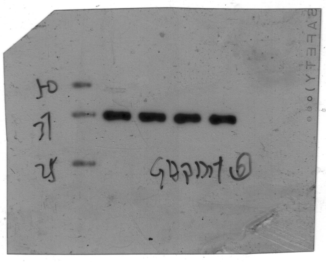


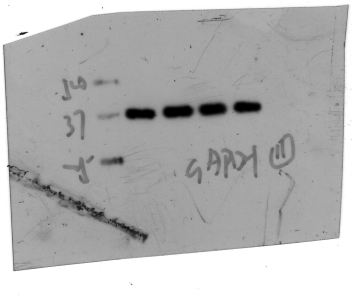

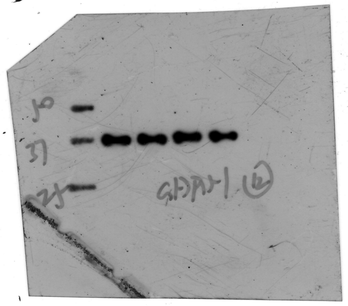
50
37
25
